# Patients’ Measurement Priorities for Remote Measurement Technologies to Aid Chronic Health Conditions: Qualitative Analysis

**DOI:** 10.2196/15086

**Published:** 2020-06-10

**Authors:** Sara Simblett, Faith Matcham, Hannah Curtis, Ben Greer, Ashley Polhemus, Jan Novák, Jose Ferrao, Peter Gamble, Matthew Hotopf, Vaibhav Narayan, Til Wykes, Remote Assessment of Disease and Relapse – Central Nervous System (RADAR-CNS) Consortium

**Affiliations:** 1 Institute of Psychiatry, Psychology and Neuroscience King's College London London United Kingdom; 2 Merck Research Labs IT Merck Sharpe & Dohme Prague Czech Republic; 3 Janssen Pharmaceuticals, LLC Titusville, NJ United States; 4 RADAR-CNS London United Kingdom

**Keywords:** qualitative analysis, patient involvement, remote measurement technology, mHealth

## Abstract

**Background:**

Remote measurement technology (RMT), including the use of mobile phone apps and wearable devices, may provide the opportunity for real-world assessment and intervention that will streamline clinical input for years to come. In order to establish the benefits of this approach, we need to operationalize what is expected in terms of a successful measurement. We focused on three clinical long-term conditions where a novel case has been made for the benefits of RMT: major depressive disorder (MDD), multiple sclerosis (MS), and epilepsy.

**Objective:**

The aim of this study was to conduct a consultation exercise on the clinical end point or outcome measurement priorities for RMT studies, drawing on the experiences of people with chronic health conditions.

**Methods:**

A total of 24 participants (16/24 women, 67%), ranging from 28 to 65 years of age, with a diagnosis of one of three chronic health conditions―MDD, MS, or epilepsy―took part in six focus groups. A systematic thematic analysis was used to extract themes and subthemes of clinical end point or measurement priorities.

**Results:**

The views of people with MDD, epilepsy, and MS differed. Each group highlighted unique measurements of importance, relevant to their specific needs. Although there was agreement that remote measurement could be useful for tracking symptoms of illness, some symptoms were specific to the individual groups. Measuring signs of wellness was discussed more by people with MDD than by people with MS and epilepsy. However, overlap did emerge when considering contextual factors, such as life events and availability of support (MDD and epilepsy) as well as ways of coping (epilepsy and MS).

**Conclusions:**

This is a unique study that puts patients’ views at the forefront of the design of a clinical study employing novel digital resources. In all cases, measuring symptom severity is key; people want to know when their health is getting worse. Second, symptom severity needs to be placed into context. A holistic approach that, in some cases, considers signs of wellness as well as illness, should be the aim of studies employing RMT to understand the health of people with chronic conditions.

## Introduction

It is estimated that by 2020, chronic health conditions will contribute to approximately 57% of the global burden of disease [1]. There is a need for innovative ways to support all these people in accessing clinical care and in managing their long-term conditions, in the context of limited resources. A case has been made for the use of mobile technology (eg, mobile phone apps and wearable technology) to provide *real-world* assessment and intervention that will both streamline clinical input and, where possible, promote independent self-management [2,3]. As an example, remote measurement technology (RMT) can gather data that may enable the early detection of worsening symptoms with the potential to offer rapid interventions. A recent systematic review identified an emergence of studies in this area [4]. In order to establish the benefits of using RMT, we need to clarify what is expected in terms of a *successful outcome*. The selection of outcomes measured is often determined by the interests of researchers, which may in part be driven by the availability of valid and reliable tools. More and more, people are advocating for the involvement of the people who receive or provide health services in translational research design [5]. The recent Academy of Medical Sciences’ report [6], *Our data-driven future in healthcare: People and partnerships at the heart of health-related technologies*, recommends that patients and the public should be active partners in agreeing on priorities for, and determining the acceptability of, data-driven technologies as part of an ongoing process.

The aim of this study was to conduct a consultation exercise on measurements of interest in RMT studies. We identified three clinical groups where there is a strong case for the potential benefits of RMT for measuring and managing recurrent and persistent chronic health conditions: major depressive disorder (MDD), a mood disorder characterized by a persistent feeling of sadness or a lack of interest in outside stimuli with a high risk of reoccurrence [7]; multiple sclerosis (MS), a relapsing or progressive demyelinating disease in which the insulating covers of nerve cells in the brain and spinal cord are damaged over time; and epilepsy, a long-term neurological condition causing frequent seizures (see Table 1). These clinical groups are varied and have different presenting characteristics, but they are all long-term, highly variable conditions that are costly to manage with largely unknown mechanisms precipitating relapse. Monitoring symptoms over time could assist with developing a better understanding of these relapse mechanisms and patterns of variability; this could potentially lead to the early identification of relapse or deterioration with the ability to intervene more quickly. Previous consultation exercises with people living with these three health conditions—MDD [8,9], MS [10], and epilepsy [11]—have identified overlapping themes that are pertinent to the introduction of mobile technologies, including the importance of self-management, prevention or prediction of symptoms, and early intervention. None of these consultations so far have asked specifically about people’s views on what might be important to measure when implementing mobile technologies. This information is crucial for being able to design systems that engage users, under the assumption that measurement of meaningful information is necessary for sustained engagement [4]. The aim of this paper is to extend previous work and focus on the use of RMT.

**Table 1:** Case examples of the use of remote measurement technology.

Health condition	Case example
Major depressive disorder (MDD)	Symptom recall for people with MDD is frequently interrupted and biased by poor cognition and dysfunctional perceptions. Reliance on self-report measures alone leads to imprecise and inefficient estimations of effects in clinical trials. Mobile technology, including wearable sensors, may allow for more momentary and continuous assessment of factors associated with MDD (eg, reduced activity or change in speech patterns and other physiology). Signs of relapse may be able to be detected before a person is fully aware of their declining mood.
Multiple sclerosis (MS)	There is emerging evidence for the reliability and validity of mobility and gait assessment using wearable activity monitoring (ie, accelerometry) for modelling relapse in MS. Use of mobile sensors, combined with more frequent (eg, daily or weekly) self-reported outcomes to contextualize changes in activity, may provide early indicators of relapse that have not been detectable in the past.
Epilepsy	Routine electroencephalogram electrode technology for monitoring health state in epilepsy cannot be implemented for more than a few days at a time. There is scope to integrate mobile technology into clinical assessment that will allow collection of continuous data to track, and possibly predict, seizure occurrence as part of daily life. Other mobile sensors (eg, wearable heart rate and activity monitors) are being investigated as alternative, potentially less obtrusive, options.

## Methods

### Design

A qualitative approach using a thematic analysis was employed to elicit views on measurement priorities from service users. Themes and subthemes were identified following grounded-theory methods.

### Context

#### Researcher Characteristics

Six focus groups were facilitated by two women—a clinical psychologist and a health psychologist—who were not involved in the participants’ clinical care.

#### Participant Characteristics

Participants were identified by convenience sampling and their eligibility to participate. Participants were included if they were over the age of 18 and had received a diagnosis of MS, epilepsy, or MDD (within the past 2 years for MDD). People with MS and epilepsy were recruited through third-sector organizations (ie, the MS Society and Epilepsy Action) and local clinics; people with MDD were recruited from a register of people who had given prior consent to be contacted about research studies and had been screened on a self-report measure of MDD: the World Health Organization's Composite International Diagnostic-Short Form [12]. Table 2 displays the characteristics of this sample in terms of their gender and age, as well as the time postdiagnosis for each health condition.

**Table 2:** Sample characteristics across the three health conditions.

Characteristic		Major depressive disorder (n=8)	Epilepsy (n=7)	Multiple sclerosis (n=9)
Gender (female), n (%)		5 (63)	5 (71)	6 (67)
Age (years), mean (SD)		51.9 (9.4)	44.4 (15.8)	43.4 (9.5)
Time postdiagnosis (years), mean (SD)		8.3 (10.3)	19.1 (16.2)	2.9 (1.6)
**Ethnicity, n (%)**				
	Caucasian	5 (63)	6 (86)	6 (67)
	Black	2 (25)	N/A^a^	N/A
	Asian	1 (13)	N/A	N/A
	Other	N/A	1 (14)	3 (33)
Theme-checking group follow-up, n (%)		6 (75)	5 (71)	5 (56)

^a^N/A: not applicable.

### Focus Group Procedure

A local research ethics committee (REC) approved these procedures (REC reference No. 16/LO/1513). All participants were screened for their eligibility to take part and, if eligible, were invited to a focus group session, for which travel expenses were covered. In this session, they first completed a consent form and a demographics questionnaire. We conducted separate focus groups for people with a diagnosis of MDD, MS, and epilepsy. For each, the main discussion was semistructured using a prespecified topic guide (available on request). The discussion was designed based on the existing literature and through consultation with health care professionals and service users to elicit ideas about what was important to people in terms of their physical and mental health and well-being (eg, whether measuring relapse was important). In the topic guide, we referenced *long-term conditions* but also focused separately on symptoms of MDD, symptoms of MS, and seizure occurrence, tailoring this to the ones most relevant to the group. The open-discussion format allowed people to share a breadth of experiences, including what was important to their health and well-being, as well as suggestions for important areas to measure using RMT. Each group’s main discussion lasted 60-120 minutes and was combined with a conversation about potential barriers and facilitators to engagement, the content of which has been published elsewhere [13-15]. We invited all participants to comment on the themes extracted from the main discussion in a second focus group. This member-checking process allowed us to validate the themes that had been extracted from the main discussion. The results from this second session were combined with the first session to add further depth and to clarify the points raised.

### Data Analysis

Focus group discussions were audio recorded and transcribed verbatim. For each health condition, analyses were conducted by two researchers working independently using the software package NVivo 11 (QSR International) [16]. Themes emerging from the data were identified in the final analysis. Disagreements in coding were resolved as a pair, and a joint decision was made about the allocation of a code to each quotation.

## Results

### Overview

A total of 24 participants, ranging from 28 to 65 years of age, took part in three focus groups; 67% (16/24) of participants were women. Of the 24 participants, 16 (67%) returned for a further member-checking session to verify the findings. This meant that six focus groups were run in total. There was a similar distribution of men and women across the focus groups. However, participants with a history of MDD were, on average, slightly older. The time spent living with the chronic health condition varied; the people with MS had, on average, been living with their condition for the shortest amount of time.

The focus groups identified several factors important to health and well-being across the three health conditions. We have divided these results into the measurement priorities important for each clinical group separately. For MDD and MS, the discussions centered around the importance of detecting signs of relapse or deterioration in health; for epilepsy, the focus was on the detection of seizures. For all groups, there was a consideration of how RMT may support well-being as well as symptoms of illness and contextual factors.

### Major Depressive Disorder

Participants were asked what was important to their health and well-being and what factors may be important to measure using RMT. They reported a plethora of possible symptoms commonly associated with relapse in MDD, including negative thoughts (ie, about being dissatisfied with themselves, unsupported, and burnt out); poor sleep; changes in appetite (ie, for some, this included experience of eating disorders); withdrawal from activities, including social activities and self-care; and anxiety, including fear of relapse.

I was thinking probably when I don’t sleep well … that’s a sign. You can get these tracker things now and I was thinking getting one myself, that’s supposed to track your sleep. I thought maybe something as simple as that might actually be helpful.

MDD participant #8, regarding poor sleep as a sign of relapse

In addition to relapse, some participants valued a focus on remission or maintenance of wellness. For measurable signs of wellness, participants had several suggestions, including being more active, such as participating in more social and other leisure activities (ie, moderate physical activity) and engaging in employment; eating well; feeling in control and actively coping with situations; feeling good about oneself; and experiencing a sense of achievement.

I like recording what keeps me well, not what makes me ill. I’d much prefer contemplating to think more positively. To think, “oh these things work.” I like to keep focused on the positive side.

MDD participant #4, regarding measuring wellness

Contextual factors that included life events, such as bereavement, problems with employment, and financial difficulties, were seen to be important to monitor. Additional physical health problems and availability of support in the context of barriers, such as social isolation, were mentioned as potentially stress-inducing contextual factors. One person mentioned the importance of tracking information that might be useful for medication management.

I could see that if um the tracking information would be useful for my doctor, to help with trying to find the right medication.

MDD participant #6, regarding the importance of tracking medication use

### Multiple Sclerosis

Participants with a diagnosis of MS also endorsed using RMT to measure and predict relapse but mostly in the context of a diagnosis of relapsing-remitting MS. For participants with a diagnosis of progressive forms of MS, relapse was less important because this did not reflect their experience of living with their condition.

The other thing I’d find useful would be to be able to sort of track how much worse I’m getting, it’s very hard to know, because it’s very gradual in a way, the deterioration I’m getting.

MS participant #8, regarding measuring deterioration

This suggests that a focus on change in severity of symptoms would still be of importance to measure when using RMT. Deterioration in mobility and gait were key symptoms highlighted. However, participants emphasized the importance of measuring additional symptoms, such as vision (ie, for some, optic neuritis was an early symptom of MS relapse), fatigue, and social functioning. Mental health was also thought to be important to measure. Participants highlighted specific times that may be associated with greater distress, including the time before their diagnosis, and periods of relief afterward. These key moments in the trajectory of people’s illness may be particularly important targets for remote measurement and intervention.

In addition to symptoms of illness, some participants spoke of the value in measuring signs of wellness, for instance, eating well and being active. Individual contextual factors such as outlook or attitude modified their experience, with active attempts to cope being potentially protective for well-being.

If there’s something that monitors everything that you’ve eaten that day and what you’ve been doing that day and then it’s like, “okay that’s been a good day,” then you’ll have that information to think, “well maybe I’ll do more of that to try and increase the amount of good days.”

MS participant #9, regarding measuring wellness

### Epilepsy

Participants with epilepsy saw the potential importance of RMT in its ability to measure the frequency of seizures, as well as preseizure symptoms or predictors. The unpredictable nature of seizure occurrence was discussed among participants, including the potential value for technology to provide more control.

I get warnings before my seizures but they’re not very long, so if I can predict it even before that, it might change the way I plan my day.

Epilepsy participant #6, regarding value of predicting seizures

Perhaps due to the uncertainty surrounding predictors of seizure, different participants raised different parameters of importance. Those most frequently mentioned included change in emotions, including anger, anxiety, and more positive emotions such as excitement, as well as altered sleep, including sleep deprivation and irregular sleep patterns. Physiological signals, such as heart rate and *brain activity* (eg, electroencephalography), were mentioned to help detect seizures.

It is important to note that some participants felt that a singular focus on seizures may be problematic. Participants spoke of epilepsy having an impact on their life in a more holistic way. Contextual factors such as effects on working life may be just as important to track as seizure frequency. These contextual factors were framed in terms of the losses that people with epilepsy experience as a result of their health condition (eg, loss of employment).

It’s actually the 23 hours of every day when you’re not having a fit, that’s the time that the epilepsy has the biggest effect.

Epilepsy participant #4, regarding importance of context

I don’t want that constant reminder when I’m having a good day.

Epilepsy participant #6, regarding importance of the ability to forget diagnosis on well days

Despite the importance of a holistic approach, the group did not think that focusing on signs of wellness would always be of help. One person stated that it might be annoying to be constantly reminded that they had a diagnosis of epilepsy on days when they felt well. This linked to a discussion that acceptance of their own health condition was hard and potentially influenced by a felt sense of stigma. The psychosocial impact of epilepsy may be important to track.

### Comparison Across Health Conditions

**Figure 1:**
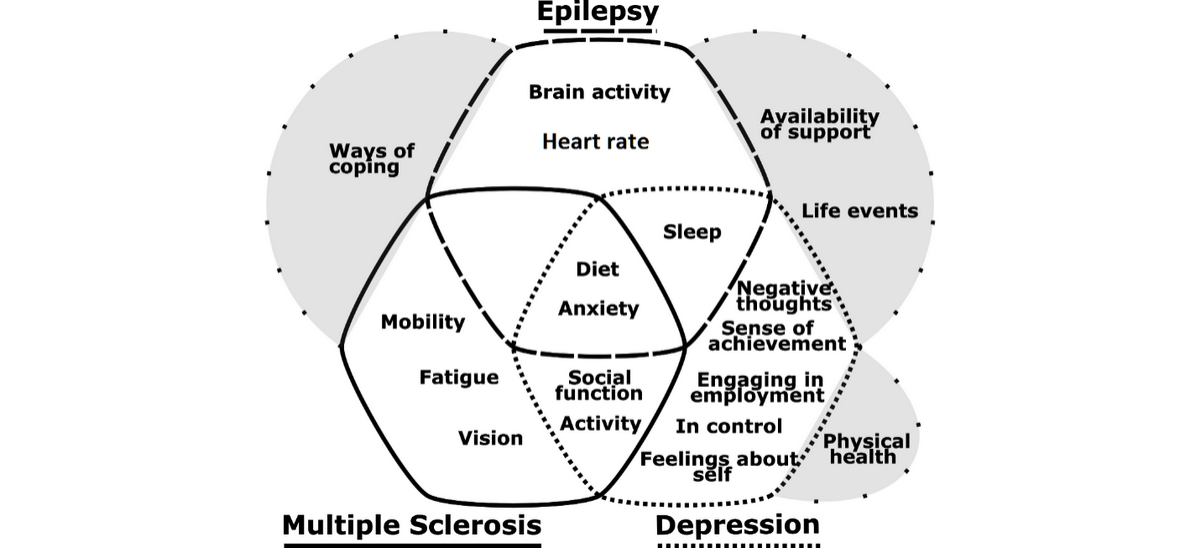
The unique and overlapping outcomes of importance for three chronic health conditions: major depressive disorder (ie, depression), epilepsy, and multiple sclerosis. Grey areas outside of the overlapping sections represent contextual factors either shared or uniquely mentioned by members of the focus groups.

From Figure 1, it is apparent that the views of people with MDD, epilepsy, and MS differed. Each group highlighted unique measurements of importance, relevant to their specific needs. Although there was agreement that remote measurement could be useful for tracking symptoms of illness, some symptoms were specific to the individual groups: for MDD this included negative thoughts; for MS it was reduced mobility and poor vison; and for epilepsy it was change in physiological parameters, such as heart rate and activity in the brain. That said, some symptoms were shared across the groups, including poor sleep (MDD and epilepsy), reduced social functioning (MDD and MS), as well as diet and anxiety (MDD, epilepsy, and MS). Measuring signs of wellness were mentioned more by people with MDD than by people with MS and epilepsy. However, there was some overlap between MDD and MS, with increased activity being important to both. Overlap also emerged when considering contextual factors, such as life events, and availability of support (MDD and epilepsy), as well as ways of coping (epilepsy and MS).

## Discussion

### Principal Findings

When participants in this consultation exercise were asked what they thought would be a *successful measurement* for the implementation of RMT, they endorsed the idea of detecting and predicting relapse (for MDD and relapsing-remitting MS) or negative change in health state (ie, deterioration for progressive forms of MS and seizure occurrence for epilepsy). Symptoms of relapse or negative change in health as described in the focus groups have been well documented; they form the basis of clinical assessment interviews and self-report tools that have been validated to measure severity of MDD (eg, the nine-item Patient Health Questionnaire) [17], MS (eg, the UK Functional Independence Measure and Functional Assessment Measure) [18], and epilepsy (eg, the Liverpool Seizure Severity Scale) [19]. These are very clearly measurements of interest for studies using RMT. If symptoms can be identified early, timely interventions may be offered, before these symptoms become more severe.

### A Holistic and Context-Specific Approach

It is important to view the conditions MDD, MS, and epilepsy both separately and holistically, meaning that we choose end points that can help us to understand people as unique individuals experiencing complex health conditions and environments. People with MDD did not only want to be monitored for symptoms of MDD, but also anxiety. In addition, they wanted to measure their physical health. This is in line with existing research on the importance of physical health as a risk factor for MDD [20-22]. For people with MS and epilepsy, the combination of measuring mental health as well as physical health emerged too. MDD and anxiety are prevalent disorders among people with both MS [23] and epilepsy [24,25], and may contribute to early signs of relapse or deterioration in the health state. Using RMT to actively measure fluctuations in mood disorder and anxiety in real time may help to gather more reliable findings. RMTs are uniquely positioned to be able to address problems with recall bias introduced when there is a delay in self-reporting of experiences.

For MDD and MS, there were discussions about maintaining *wellness* and what this looked like, most commonly, in terms of increased activity, positive social functioning, and access to support. There may be an argument for including real-time measures of well-being (eg, the Warwick-Edinburgh Mental Well-being Scale) [26] and quality of life (eg, the EuroQol five-dimension questionnaire) [27] for RMT studies conducted for these groups. Passive measures of functioning gained through an analysis of mobile phone usage and wearable devices (eg, call logs and step counts) may also be of value. For people with epilepsy, there was little focus on maintaining *wellness*; people spoke about their illness being out of their control with unpredictable triggers in terms of how they were living their life. Difficulties establishing triggers for seizures has been a well-documented finding within the previous literature [28]. For people with epilepsy, being able to receive a warning of their seizure early was of most importance to them. This highlights a difference between the needs of people with epilepsy compared to the two other chronic health conditions.

### Strengths and Limitations

The strengths of this study include the opportunity for an open and in-depth discussion with people who have first-hand experience of living with one of three chronic health conditions. This enabled a rich exploration of the health measurements of importance and allowed us to identify similarities and differences between the groups. The employed member-checking methods allowed validation of the results generated from the main discussion. Given the qualitative approach, we are limited in our ability to quantify the numbers of people wanting to measure specific outcomes or to run any statistical analyses to explore the significance of group differences, including factors such as diagnosis, age, ethnicity, and other characteristics not quantified, like the previous use of mHealth resources and income. This work has generated ideas that will inform the design of RMT studies. These RMT studies will test the relationships between the measurements of interest, including those identified in these focus groups.

### Conclusions

In this consultation exercise, we identified measurements of importance when using RMT for three chronic health conditions: MDD, MS, and epilepsy. This is a unique study that puts patients’ views at the forefront of the design of a clinical study employing novel digital resources. We draw the following conclusions. First, in all cases, measuring symptom severity is key; people want to know when their health is getting worse. Second, symptom severity needs to be placed in context. When monitoring someone with a mental health condition such as MDD, social and physical health outcomes should also be considered, and vice versa for physical health conditions such as MS and epilepsy. A holistic approach that considers situational and attitudinal factors (eg, employment, social status, acceptance of health condition, eating patterns, and ways of coping) will enable a more complete picture of how unwell a person is feeling. For some people with MDD and MS, factors that maintain well-being are just as important as factors that contribute to relapse or deterioration in health status.

## Abbreviations

EFPIA: European Federation of Pharmaceutical Industries and Associations
IMI: Innovative Medicines Initiative
MDD: major depressive disorder
MS: multiple sclerosis
NHS: National Health Service
NIHR: National Institute for Health Research
RADAR-CNS: Remote Assessment of Disease and Relapse – Central Nervous System
REC: research ethics committee
RMT: remote measurement technology
